# Adherence, control of cardiometabolic factors and therapeutic inertia in patients with type 2 diabetes in the primary care setting

**DOI:** 10.1002/edm2.320

**Published:** 2021-12-28

**Authors:** Domingo Orozco‐Beltrán, Sergio Cinza‐Sanjurjo, José Escribano‐Serrano, Flora López‐Simarro, Gonzalo Fernández, Antón Gómez García, Karine Ferreira de Campos, Marta Cedenilla Horcajuelo

**Affiliations:** ^1^ Medicina de Familia Departamento de Medicina Clínica Universidad Miguel Hernandez San Juan de Alicante Spain; ^2^ Medicina de Familia Centro de Salud Porto do Son. Porto do Son La Coruña Spain; ^3^ Medicina de Familia Centro de Salud San Roque. San Roque Cádiz Spain; ^4^ Medicina de Familia ABS Martorell Urbano. Institut Català de la Salut Martorell Spain; ^5^ Global Medical and Scientific Affairs MSD Spain Madrid Spain

**Keywords:** adherence, diabetes, dyslipidemia, therapeutic inertia

## Abstract

**Introduction:**

Studies on treatment adherence to glucose‐lowering drugs among patients with type 2 diabetes (T2D) including concomitant treatment for other cardiovascular risk factors are scarce. We aimed to estimate the prevalence of good adherence to all medications used to control diabetes, hypertension and dyslipidemia and to analyse cardiometabolic control and its associated factors in T2D patients in the primary care (PC) setting.

**Methods:**

Observational, retrospective study conducted in adult patients with T2D who were followed in the PC setting in Spain. Patients were classified as adherent in a particular category if the summary of the proportion of days covered (PDC) for a particular medication category was ≥80% and were considered globally adherent if the PDC was ≥80% for each of the 3 medication categories.

**Results:**

A total of 457 evaluable patients were recruited, among which 321 patients (70.3%, 95% CI 65.8 to 74.4) were adherent to the three drug categories. The proportion of patients controlled for the 3 cardiometabolic risk factors was 31% according to the contemporary clinical practice guideline criteria, 58% according to investigator judgment and 36% when the objective for HbA1c was individualized. In a multivariate analysis, presenting comorbidities was associated with a lower likelihood of showing adequate control of dyslipidemia (odds ratio [OR] 0.25, 95% CI, 0.16–0.40) and the three cardiometabolic factors as a whole (OR 0.43, 95% CI 0.26–0.70). In a post hoc analysis, therapeutic inertia was found to be greater for dyslipidemia and hypertension than for T2D.

**Conclusions:**

Despite a relatively high adherence to all medications for treating diabetes, hypertension and dyslipidemia in patients with T2D in the PC setting in Spain, the control of cardiometabolic risk factors as a whole is far from optimal. This could be related, at least in part, to the high frequency of comorbidity of these patients.

## Graphical abstract

Information on adherence to the whole complex regimen that patients with diabetes require for the treatment of their disease and the management of cardiovascular risk factors is scanty. Despite a relatively high adherence to all medications for treating diabetes, hypertension and dyslipidemia in patients with type 2 diabetes in the primary care setting in Spain, the control of cardiometabolic risk factors as a whole is far from optimal. This results could be related, at least in part, to the high frequency of comorbidity of these patients.

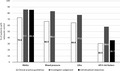

## 1 INTRODUCTION

Adherence to medication is defined as the extent to which patients take medications as prescribed by their health care professionals.^1^ According to the World Health Organization, poor adherence in individuals with chronic illnesses that require long‐term treatment is a worldwide problem with rates that average 50%. Poor adherence is associated with poor health outcomes and increased health care costs and compromises health system effectiveness overall.^2^ Poor adherence is a multifactorial phenomenon involving socioeconomic, health care system, patient‐related, disease‐related and treatment‐related factors.^1^, ^3^ Among disease‐ and treatment‐related factors, the presence of comorbid conditions and the complexity of treatment regimens are recognized factors that can negatively affect treatment adherence.^1^, ^3^

Diabetes is a chronic disease that requires a stepwise treatment approach, with the treatment becoming more complex as the disease progresses.^4^ Thus, among ambulatory physician practices in the USA in 2012, in 58% of the visits for diabetes, patients were receiving two or more antihyperglycemic medications.^5^ This situation is further complicated by the fact that, consistent with guideline recommendations,^6^ patients with diabetes require treatments for the management of cardiovascular risk factors, including hypertension and dyslipidemia. When accounting for all prescribed medications, in 2000, the annual proportion of primary care visits of patients with diabetes listing at least 5 prescription medicines was 30%,^7^ with blood pressure and lipid‐lowering drugs as major contributors to this increasing complexity of pharmacologic regimens.^8^ This situation may explain in part why treatment adherence in patients with diabetes is poor, being one of the chronic diseases with the lowest rate of adherence.^9^, ^10^ Overall, good adherence to diabetes treatment ranges from 31% to 80%,^9^, ^10^, ^11^, ^12^, ^13^, ^14^, ^15^, ^16^ depending on the study design, prescribed drug, extent of the follow‐up and/or definition of adherence. Poor adherence to antihyperglycemic treatments has been associated with poorer diabetes control,^12^, ^17^ increased risk of all‐cause hospitalization^15^ and all‐cause mortality.^15^, ^18^

Despite all the available information on adherence to antihyperglycemic agents, much less is known about adherence to the whole complex regimen that patients with diabetes require for the treatment of their disease and the management of cardiovascular risk factors. Lopez‐Simarro et al.^13^ reported a rate of nonadherence among 320 patients with diabetes seen in primary care in Spain of 36%, 38% and 32% for medications for diabetes, hypertension and dyslipidemia, respectively. Additionally, in the same study, the proportion of patients with T2D with good control of HbA1c, blood pressure and LDL‐cholesterol was 62.5%, 40.9% and 35.9%, respectively.^19^ Lower rates of nonadherence were reported by Ho et al.^15^ in patients with diabetes in a US managed care organization (20%, 19% and 25% for antihyperglycemics, antihypertensives and statins, respectively). Many clinical guidelines and expert committees recommend the individualization of glycaemic targets and treatment decisions in the management of type 2 diabetes (T2D) depending on patient's preferences and characteristics, such as frailty or comorbid conditions, with special interest in the presence of cardiovascular or renal disorders.^20^, ^21^ This approach has the goal of reducing complications and maintaining quality of life in the context of comprehensive cardiovascular risk management and patient‐centred care. We performed a retrospective study whose primary objective was to estimate the prevalence of good adherence to all medications used to control diabetes, hypertension and dyslipidemia in patients with T2D attending PC centres. Secondary objectives included analysing adherence within each of the therapeutic groups; comparing the levels of HbA1c, blood pressure and LDLc between adherent and nonadherent patients; and comparing the proportion of patients controlling these three risk factors between adherent and nonadherent patients in this population. A post hoc objective was to evaluate therapeutic inertia for the treatment of diabetes, hypertension and dyslipidemia and to analyse associated factors.

## 2 MATERIALS AND METHODS

### 2.1 Study design, setting and patients

This was an observational, retrospective study. The study was approved by the Ethics Committee of each participating site. Eighty primary care physicians throughout Spain recruited patients consecutively during a single inclusion visit. The index date was established as the date 365 days before the date of the inclusion visit. To be included in the study, patients had to be 18 years or older; have been diagnosed with T2D; be followed by a primary care physician; be prescribed oral antihyperglycemic drugs (OAHDs), antihypertensive drugs for the treatment of hypertension and lipid‐lowering drugs for the treatment of dyslipidemia for at least 12 months; and not have changed their residence in the last 12 months. Patients were excluded if they were unable to provide their written informed consent; were dependent; had been participating in a clinical trial at any time during the 1‐year study period; had a psychiatric disorder other than a depressive or anxiety disorder; had a severe or terminal disease; were receiving insulin or glucagon‐like peptide 1 receptor agonists; or became pregnant or were diagnosed with ketoacidosis, malnutrition‐associated diabetes, drug‐induced diabetes or gestational diabetes during the 1‐year study period.

### 2.2 Medications and estimation of treatment adherence

Information on medication received was obtained from the Spanish electronic medical prescription system (eReceta). eReceta was initiated in 2004 and was implemented across all autonomous regions in Spain; the system relies on the patient's electronic health card and the link of the card to his/her medical records in several databases. Medication adherence was calculated as the proportion of days covered (PDC). The PDC was based on the filled e‐prescriptions during the 1‐year study period for each of the three categories of medications: OAHDs, antihypertensives and lipid‐lowering drugs. To calculate the PDC, we estimated the total days of supplies from the first refill to the last refill during the 1‐year observation period, divided by the total days of the treatment interval; the treatment interval was defined as the time elapsed from the date of the first refilled e‐prescription to the end of the observation period regardless of whether the patient was maintained on the first drug prescribed or was switched to another drug or, if the drug was discontinued by the physician, until the date the primary care physician recommended discontinuing the drug. The resulting figure was multiplied by 100 to estimate the percentage of PDC. The PDC was averaged for all drugs within a category. Patients were categorized as adherent to a particular category if the summary PDC for that category was 80% or greater. Patients were considered globally adherent if the PDC was ≥80% for each of the three medication categories.

### 2.3 Demographics, clinical assessments and definition of disease control and therapeutic inertia

Demographics and clinical data were obtained from the patient's electronic clinical record or, if not available, at the time of the inclusion visit. Demographics included age, sex, race and household status. Clinical data included data from the physical examination (weight, height and body mass index) and diabetes‐related complications (retinopathy, nephropathy, neuropathy and diabetic foot) and comorbidities (coronary artery disease, heart failure, peripheral occlusive arterial disease, cerebrovascular disease, depression, osteoarthritis, chronic obstructive pulmonary disease and others); regarding diabetes‐related complications and comorbidities, whether they were present at the time of index date or had occurred during the 1‐year observation period was recorded. Information on the degree of cardiometabolic control (ie HbA1c, blood pressure and LDLc) was obtained from the electronic clinical record if it was recorded within 3 months before the inclusion visit or was measured at the time of the inclusion visit by measuring blood pressure and/or performing blood extraction for laboratory analysis.

Glycaemic control was evaluated according to the clinical practice guidelines and based on individualized criteria. Patients were considered controlled according to the clinical practice guideline criteria if HbA1c was <7.0%^22^ and based on individualized criteria: if patients were younger than 75 years old with less than 10 years of diabetes duration and had no diabetes‐related comorbidities (ie coronary heart disease, heart failure or occlusive peripheral arterial disease) or complications (ie retinopathy, nephropathy, neuropathy or diabetic foot), they were considered controlled if HbA1c was <6.5%; and if patients met any of the latter criteria, they were considered controlled if HbA1c was <7.5%. The guideline‐based criteria for categorizing patients as having adequate disease control in terms of hypertension and dyslipidemia were as follows: systolic blood pressure <140 mm Hg and diastolic blood pressure <90 mm Hg^22^ and LDLc <100 mg/dl for primary prevention and <70 mg/dl for secondary prevention (as per contemporary guidelines).^23^ In addition, physicians were asked to assess whether, regardless of the actual values of patients' cardiometabolic parameters and based on the clinical characteristics, they considered that the patient was controlled for each of these 3 factors.

We considered that there was therapeutic inertia if a patient was adequately controlled based on the physician's criteria but was not controlled according to the cardiometabolic parameters as previously described.^24^

### 2.4 Statistical analysis

To achieve 5% statistical precision in the estimation of a population proportion with an asymptotic normal 95% confidence interval and assuming a nonadherence proportion of 50%, it would be necessary to include 384 patients in the study; assuming a 10% rate of non‐evaluable patients, it would be necessary to recruit 428 subjects.

The characteristics of the recruited population are presented with means and standard deviations for continuous variables and absolute and relative frequencies for qualitative variables. To describe the prevalence, the point estimate and the corresponding 95% confidence interval are provided. In the bivariate analysis, characteristics of the adherent and nonadherent patients were compared using Student's *t* test or the Mann‐Whitney test for continuous variables and the chi‐squared test or Fisher's exact test for categorical variables.

To explore the factors associated with global adherence, a multiple logistic regression analysis was performed. The dependent variable was global adherence, and the independent variables were age, BMI, HbA1c, LDLc, blood pressure, number of medicines, number of diabetes‐related complications and number of comorbidities. To explore whether adherence was associated with the control of the cardiometabolic risk factors, four multiple logistic regression analyses were performed using the control of each cardiometabolic factor or, in the fourth model, the control of the three factors as dependent variables; the independent variables were adherence (adherence with each pharmacologic category for the control of each individual cardiometabolic risk factor and global adherence for the multivariate analysis of the global control of the risk factors), age (≤75 years and >75 years), sex, BMI (categorized as ≥30 and <30), the presence of diabetes complications before the index period and the presence of comorbid conditions before the index period.

To explore factors associated with therapeutic inertia for each of the cardiometabolic risk factors, we performed four multiple logistic regression analyses using the presence of therapeutic inertia for each cardiometabolic factor (in the case of T2D using the clinical practice guideline and individualized criteria for defining disease control) as dependent variables; the independent variables were age; sex; BMI; diabetes duration; HbA1c; LDL; systolic blood pressure; diastolic blood pressure; number of daily pills for treating T2D, hypertension and dyslipidemia; total number of pills; whether the patients were living alone; the presence of diabetes complications before the index period; the presence of comorbid conditions before the index period; the presence of depression before the index period; the presence of osteoarthritis before the index period; and the presence of chronic obstructive disease before the index period. For the selection of factors, we used a stepwise forward selection based on Akaike's information criterion.

All analyses were performed with SPSS v.18.0 (IBM Corp.) or R 3.4.3 (The R Project for Statistical Computing), and the results were considered significant at *p *< .05.

## 3 RESULTS

### 3.1 Patient disposition and characteristics

A total of 475 patients were recruited by thirty‐six participating sites from four autonomous communities of Spain (Galicia [Northwest], Andalucia [South], Cataluña [Northeast] and Valencia [East]) from May 2016 to November 2016. Eighteen (3.8%) patients were excluded from the analysis because either they did not meet inclusion criteria or they did not sign the informed consent, leaving 457 evaluable patients who were included in the analyses.

The demographic and clinical characteristics of the patients are presented in Table 1. Patients were elderly and almost evenly distributed according to sex. The mean T2D duration was 7.3 years, and the mean BMI was 30.9 kg/m^2^. On average, patients were receiving over 4 oral medications for the treatment of T2D, hypertension and dyslipidemia, and overall, they were receiving almost 10 drugs on average (Table 1). The most frequent T2D‐associated complication was nephropathy (12%), and the most frequent comorbidities were osteoarthritis (44%) and, to a lesser extent, depression (19%) and coronary artery disease (18%).

**TABLE 1:** Demographic and clinical characteristics at the inclusion visit

Characteristic	*N* with available data	Mean (SD)/median (IQR) or proportion of patients
Age (years), mean (SD)/median (IQR)	457	71.2 (9.4)/ 72 (66.0–78.0)
Sex (women), *n* (%)	457	217 (47.5%)
Race (Caucasian), *n* (%)	457	454 (99.3%)
Weight (kg), mean (SD)/median (IQR)	457	80.2 (14.1)/ 78.5 (70.1–88.0)
Body mass index (kg/m^2^), mean (SD)/median (IQR)	457	30.9 (4.7)/ 30.6 (27.8–33.3)
Diabetes duration (years), mean (SD)/median (IQR)	457	7.3 (5.4)/ 6.6 (3.4–8.8)
Cardiometabolic risk factors (CMRF), mean (SD)/median (IQR)		
HbA1c (%)	456	6.7 (0.9)/ 6.5 (6.1–7.0)
LDLc (mg/dl)	451	85.8 (25.8)/ 84.0 (69.0–97.0)
Systolic blood pressure (mm Hg)	457	133.2 (13.7)/ 132.0 (124.5–140.0)
Diastolic blood pressure (mm Hg)	456	74.9 (9.2)/ 75.0 (70.0–81.0)
Patients with CMRF adequately controlled according to the clinical practice guidelines	451	35.7%
Patients with CMRF adequately controlled according to physician judgment	452	58.4%
Pharmacologic treatment (number), mean (SD)/median (IQR)		
Oral antihyperglycemic agents (OAHs)	457	1.4 (0.7)/ 1.0 (1.0–2.0)
Antihypertensives (AHTs)	457	1.6 (0.8)/ 1.0 (1.0–2.0)
Lipid‐lowering drugs (LLDs)	457	1.1 (0.4)/ 1.0 (1.0–1.0)
OADs + AHTs + LLDs	457	4.1 (1.2)/ 4.0 (3.0–5.0)
Other pharmacologic treatments	457	4.3 (3.6)/ 3.0 (2.0–6.0)
All pharmacological treatments	457	9.8 (4.3)/ 9 (6–12)
Diabetes‐associated complications, *n* (%)		
Retinopathy	432	23 (5.3)
Nephropathy	437	55 (12.6)
Neuropathy	434	11 (2.5)
Diabetic foot	443	0 (0.0)
Comorbidities		
Osteoarthritis	451	199 (44.1)
Depression	454	86 (18.9)
Coronary artery disease	451	82 (18.1)
Cerebrovascular disease	451	38 (8.4)
Chronic obstructive pulmonary disease	448	37 (8.3)
Heart failure	450	27 (6.0)
Peripheral occlusive arterial disease	448	26 (5.8)

Abbreviations: CMRF, cardiometabolic risk factors; IQR, interquartile range; LDLc, low‐density lipoprotein cholesterol; SD, standard deviation.

### 3.2 Adherence to medications for the management of cardiometabolic risk factors

**FIGURE 1:**
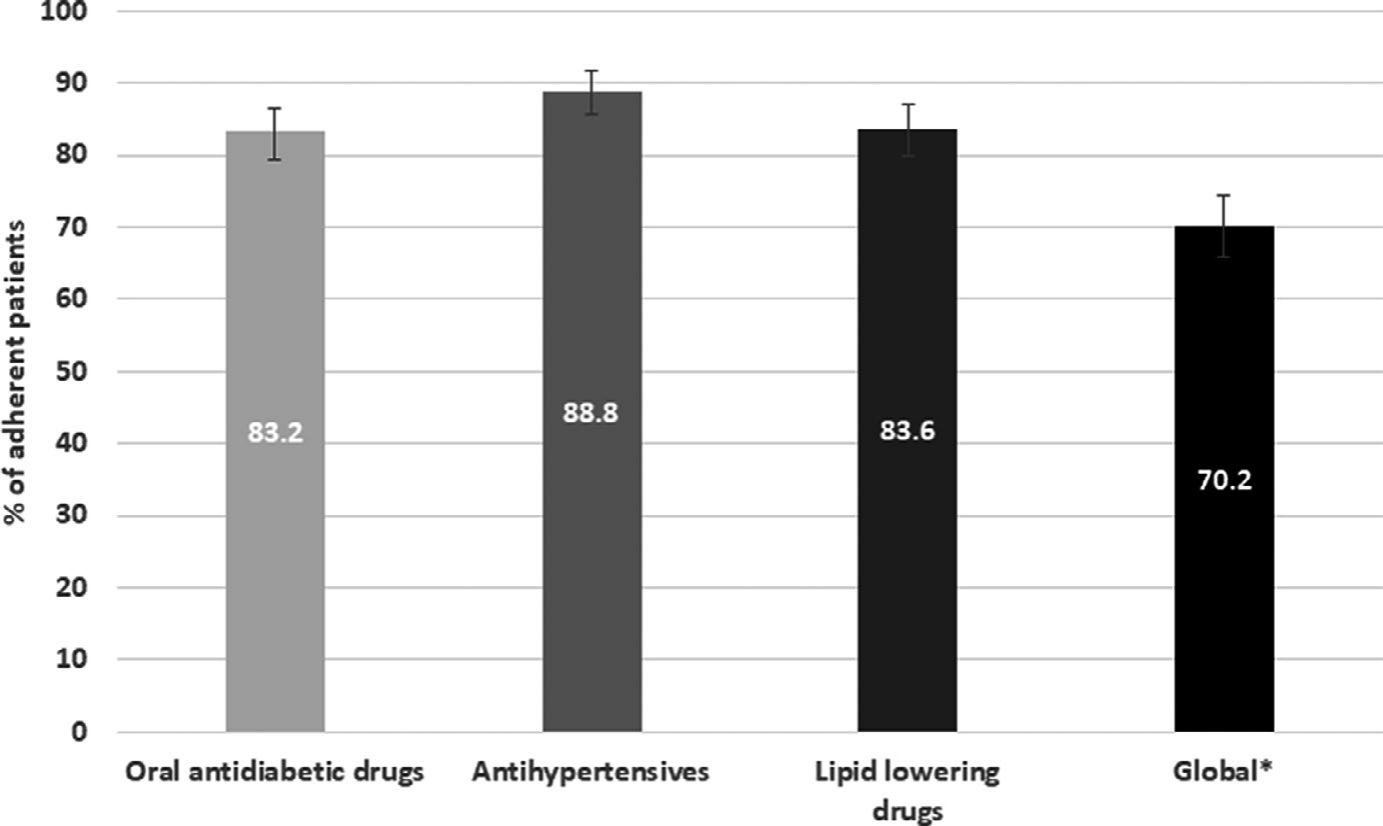
Patients with type 2 diabetes adherent to oral medications for diabetes, hypertension and dyslipidemia. ^†^Patients were considered overall adherent if the percentage of days covered was ≥80% for each of the 3 medication categories

Overall, 321 patients (70.2%, 95% CI 65.8 to 74.4) were adherent to all three drug categories, namely oral antihyperglycemic agents, antihypertensives and lipid‐lowering drugs; of the remaining patients, 86 (18.8%) were adherent to two pharmacologic categories, 33 (7.2%) were adherent to only one pharmacologic category, and 17 (3.7%) were completely nonadherent. Adherence was over 80% for each individual pharmacologic category (Figure 1).

### 3.3 Control of cardiometabolic risk factors and their association with adherence

**FIGURE 2:**
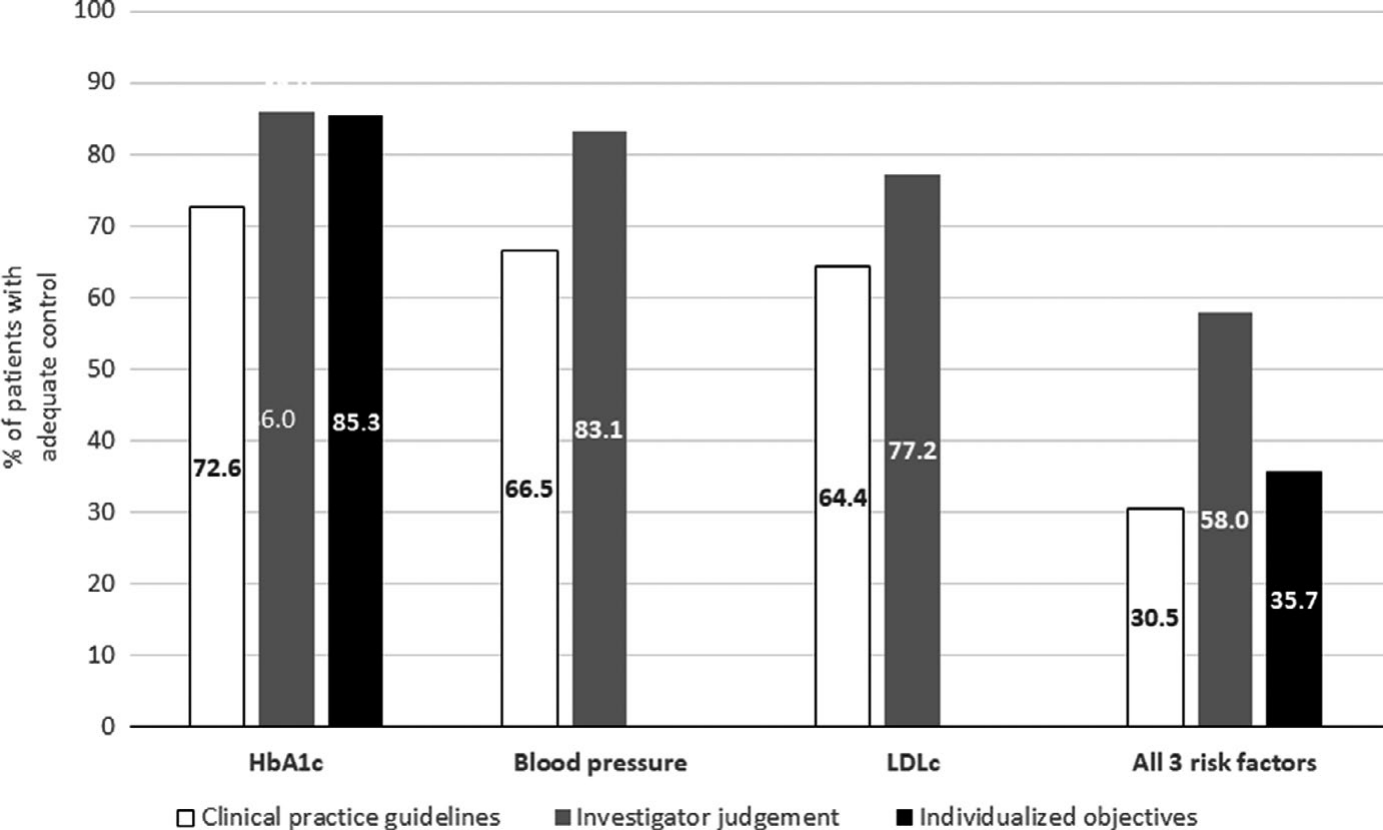
Patients with adequate control of cardiometabolic risk factors

The proportions of patients who were considered to have controlled cardiometabolic risk factors according to the clinical practice guideline criteria, investigator judgment or individualized objectives for HbA1c are presented in Figure 2. Regardless of the cardiometabolic risk factor, the proportion of patients with a particular risk factor controlled was higher according to investigator judgment than according to the clinical practice guideline (CPG) criteria. The proportion of patients with all three cardiometabolic risk factors controlled was 31% according to the clinical practice guideline criteria, 58% according to investigator judgment, and 36% when the HbA1c objective was individualized.

In the bivariate analysis (Table 2), there was no difference between adherent and nonadherent patients regarding demographic and clinical characteristics. The only difference regarding the cardiometabolic risk factors between globally adherent and nonadherent patients was the level of LDLc, which was significantly lower among adherent patients (84 vs. 91 mg/dl, *p *= .034). Similarly, there was no difference between adherent and nonadherent patients regarding the proportion of patients with adequate control of cardiometabolic risk factors evaluated with either the CPG criteria or investigator judgment (Table 2).

**TABLE 2:** Bivariate analysis of the demographic and clinical characteristics of global adherent and nonadherent patients

Outcome	Adherent *N* = 321	Nonadherent *N* = 136	*p*‐value
Demographic and key clinical characteristics			
Age (years), mean (SD)	71.4 (9.3)	71.9 (9.6)	.593
Sex (women), n (%)	156 (48.8)	60 (44.9)	.528
Diabetes duration (years), mean (SD)	7.4 (5.2)	7.4 (5.2)	.531
Weight (kg), mean (SD)	80.6 (14.6)	79.0 (12.7)	.256
BMI (kg/m^2^), mean (SD)	31.0 (4.8)	30.4 (4.4)	.203
Comorbidities (Yes), n (%)	92 (29.1)	39 (39.5)	.927
Complications (Yes), n (%)	56 (17.4)	23 (17.4)	.781
Total number of pills, mean (SD)	4.1 (1.9)	4.0 (1.1)	.488
Cardiometabolic parameters, mean (SD) values at the inclusion visit			
HbA1c (%)	6.7 (0.8)	6.7 (1.0)	.546
Systolic blood pressure (mm Hg)	132.6 (13.4)	134.5 (14.6)	.192
Diastolic blood pressure (mm Hg)	74.7 (9.4)	74.9 (10.7)	.795
LDLc (mg/dl)	84.1 (23.2)	90.5 (31.1)	.034
Adequate control at the inclusion visit according to CPG criteria, n (%)			
HbA1c <7%	227 (71.2)	103 (76.1)	.280
Blood pressure <140/90 mm Hg	214 (66.9)	88 (65.7)	.804
LDLc <100 or 70 mg/dl	205 (64.7)	80 (63.0)	.739
Adequate control at the inclusion visit as judged by the investigator, n (%)			
HbA1c	276 (86.5)	113 (84.3)	.541
Blood pressure	267 (83.7)	109 (81.3)	.542
LDLc	248 (77.7)	98 (75.4)	.590

Abbreviations: CPG, clinical practice guideline; LDLc, low‐density lipoprotein cholesterol; SD, standard deviation.

**FIGURE 3:**
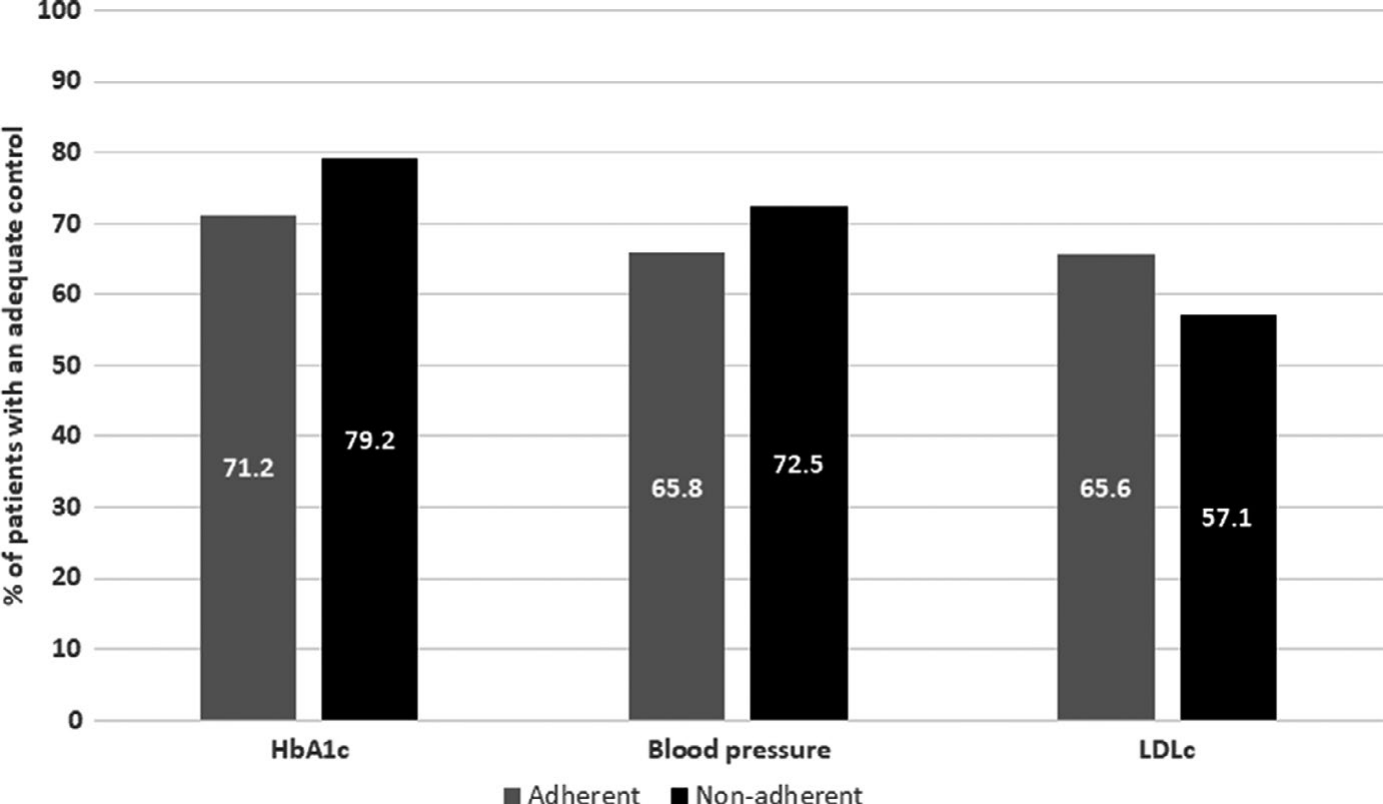
Patients with adequate control of cardiometabolic risk factors according to adherence to specific treatments. Note: adherence refers to a specific treatment (eg HbA1c adherence refers to oral antidiabetic agents)

In the multivariate analysis (Table 3), global adherence was not associated with the global control of cardiometabolic risk factors. When the control of cardiometabolic risk factors was evaluated according to the adherence of the corresponding specific pharmacologic category, there were no differences in the control of any cardiometabolic risk factor between adherent and nonadherent patients (Figure 3); the same was applicable when global adherence to the three medication categories was considered (Table 2). Regarding other factors, an age equal to or younger than 75 years was associated with a higher likelihood of showing adequate control of blood pressure but a lower likelihood of showing adequate control of dyslipidemia; being obese was associated with a lower likelihood of showing adequate control of blood pressure but a higher likelihood of showing adequate control of dyslipidemia; and presenting with comorbidities was associated with a lower likelihood of showing adequate control of dyslipidemia and the three cardiometabolic factors as a whole (Table 3).

**TABLE 3:** Multivariate analysis of factors associated with adequate control of cardiometabolic risk factors in patients with type 2 diabetes

	HbA1c	Blood pressure	LDLc	HbA1c + Blood Pressure + LDLc
Age (>75)	0.84 (0.53–1.33)	**1.83** (1.19–2.82)**	**0.61** **(0.39–0.96)** *	0.82 (0.53–1.26)
Sex (female)	1.24 (0.80–1.92)	0.83 (0.55–1.27)	1.41 (0.91–2.16)	1.16 (0.76–1.75)
BMI (<30)	1.21 (0.78–1.86)	**0.55** (0.36–0.84)**	**1.61** **(1.06–2.47)** *	1.02 (0.68–1.53)
Complications (No)	0.60 (0.35–1.05)	0.79 (0.47–1.34)	0,88 (0.50–1.53)	0.71 (0.40–1.26)
Comorbidities (No)	1.62 (0.97–2.72)	0.77 (0.49–1.21)	**0.25** (0.16–0.40)***	**0.43** (0.26–0.70)**
Adherence^†^ (No)	0.62 (0.34–1.15)	0.70 (0.35–1.39)	1.59 (0.91–2.78)	0.85 (0.55–1.31)

Note

All figures are odds ratios and their corresponding 95% confidence intervals. The reference category appears between brackets.

* *p*<0.05, **p<0.01, ***p<0.001.

^†^ Adherence with each pharmacologic category for the control of each cardiometabolic risk factor and global adherence for the multivariate analysis of the global control of the risk factors.

Bold values are used to highlight statistically significant results.

When control was evaluated based on individualized criteria for HbA1c, among those who had adequate control of the three risk factors, 30.8% were globally nonadherent compared with 69.2% who were globally adherent.

### 3.4 Therapeutic inertia

**FIGURE 4:**
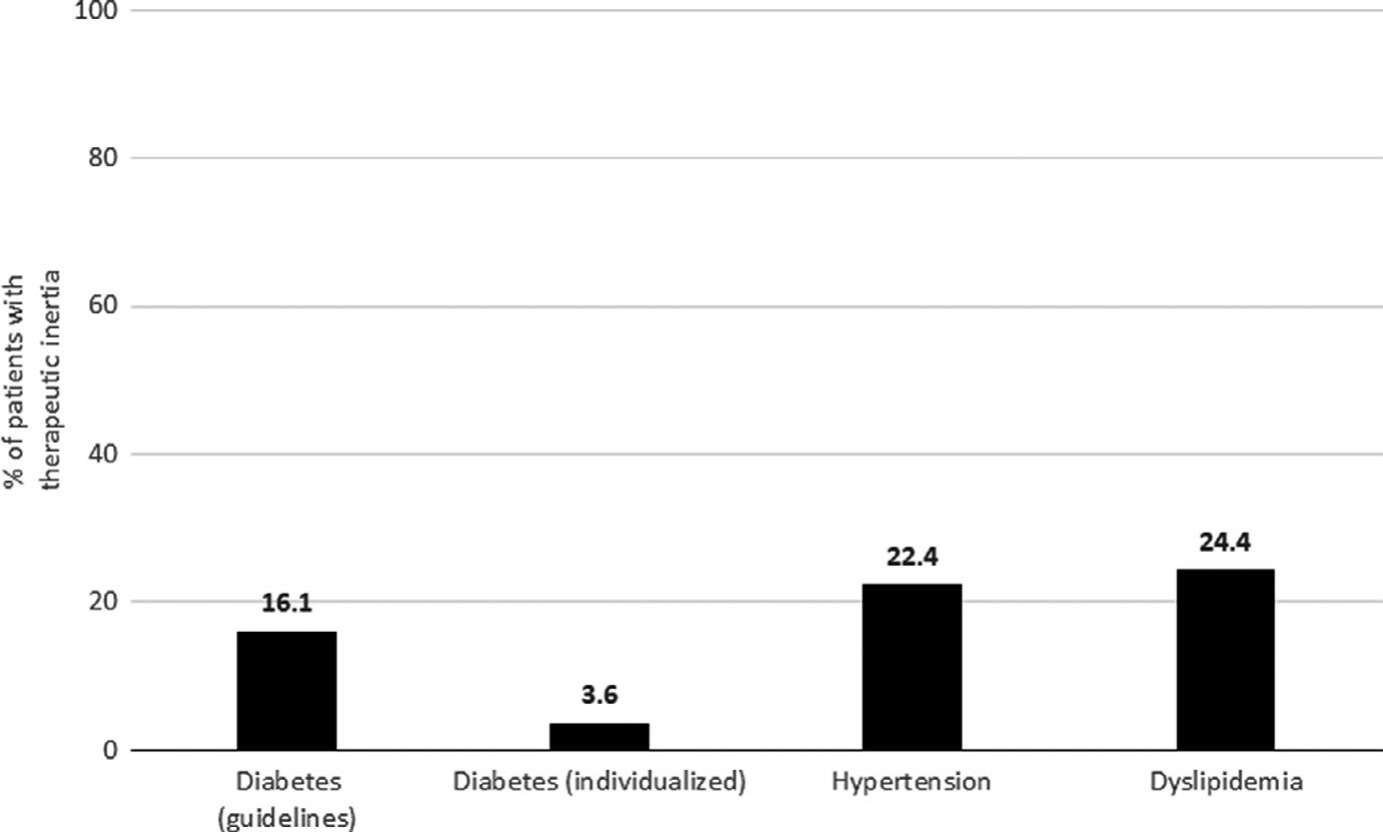
Therapeutic inertia in patients with diabetes for diabetes, hypertension and dyslipidemia. Therapeutic inertia exists if a patient was adequately controlled based on the physician criteria but he/she was not controlled according to the actual cardiometabolic parameters. When interpreting the actual cardiometabolic parameters, patients were considered to have controlled diabetes (a) according to the clinical practice guidelines if HbA1c was <7.0%; (b) based on individualized criteria, if patients were younger than 70 years old with less than 10 years of diabetes duration and had no diabetes‐related comorbidities (ie coronary heart disease, heart failure or occlusive peripheral arterial disease) or complications (ie retinopathy, nephropathy, neuropathy or diabetic foot), they were considered controlled if HbA1c was <6.5%; and if patients met any of the latter criteria, they were considered controlled if HbA1c was <7.5. The criteria for categorizing patients as having adequate disease control regarding hypertension and dyslipidemia were as follows: systolic blood pressure <140 mm Hg and diastolic blood pressure <90 mm Hg (19); and an LDLc <100 mg/dl for high‐risk patients and <70 mg/dl for very high‐risk patients

Therapeutic inertia was greater for dyslipidemia and hypertension than for T2D (Figure 4). Therapeutic inertia for the treatment of T2D differed depending on disease control definition according to the clinical practice guideline criteria or based on individualized criteria (Figure 4); from the 63 subjects who exhibited therapeutic inertia regarding T2D treatment based on the clinical practice guideline criteria for HbA1c, only 14 (22%) continued to exhibit therapeutic inertia when disease control was defined using individualized criteria for HbA1c.

In the multivariate analyses, we did not find any factor associated with therapeutic inertia for the treatment of T2D when individualized criteria for HbA1c were considered. Age was directly associated with a higher likelihood of therapeutic inertia for the treatment of T2D based on clinical guideline criteria and hypertension and inversely associated with inertia in the management of dyslipidemia (Table 4). The presence of diabetes‐related comorbidities prior to the index period was associated with an almost fivefold increase in the likelihood of exhibiting therapeutic inertia for the treatment of dyslipidemia, while each point increase in body mass index was associated with an 8% reduction in the likelihood of exhibiting therapeutic inertia in this regard.

**TABLE 4:** Multivariate analysis of factors associated with therapeutic inertia in patients with type 2 diabetes

	HbA1c (guidelines)	Blood pressure	LDLc
Age	1.04 (1.01–1.07)	1.03 (1.00–1.07)	0.95 (0.93–0.98)
Sex (female)	1.38 (0.79–2.40)^†^	0.74 (0.44–1.25)^†^	1.67 (0.96–2.91)^†^
Body mass index	–	–	0.92 (0.86–0.98)
Diabetes‐related complications prior to the index period (No)	–	1.99 (1.08–3.66)	–
Diabetes‐related comorbidities prior to the index period (No)	–	–	4.98 (2.80–8.84)
History of COPD (No)	–	0.34 (0.11–1.04)*	–
ROC area	0.62 (0.54–0.70)	0.64 (0.57–0.71)	0.72 (0.66–0.78)

Note

All figures are odds ratios and their corresponding 95% confidence intervals. The reference category appears between brackets; the lack of reference category means that the factor was included as a continuous variable.

^†^ These variables were maintained in the model because they were considered confounding factors.

## 4 DISCUSSION

Our results show that in patients with T2D treated with oral antihyperglycemic agents, adherence to all medications for treating T2D, hypertension and dyslipidemia is relatively high. However, although the control of individual risk factors is relatively high, the proportion of patients showing adequate control of all three risk factors is low.

We found an overall adherence to all medications for treating diabetes, hypertension and dyslipidemia of 70%. Unfortunately, we are aware of only one study providing global figures for adherence. In a study conducted in the USA, Ho et al.^15^ reported a slightly higher overall rate of adherence to the three types of medications of 79% using a similar definition but in a different population (namely patients were not necessarily exhibiting hypertension and dyslipidemia, and they could be receiving insulin).^15^ Lopez‐Simarro et al.^13^ in a similar setting as that in our study, reported rates of nonadherence for the individual components of the treatment of cardiometabolic risk factors that were higher than those found in our study. They found that 36%, 38% and 32% of participants were nonadherent to medications for the treatment of diabetes, hypertension and dyslipidemia, respectively,^13^ while our corresponding figures were 17%, 11%, and 15%, respectively. In addition, in the same study, patients with T2D with good control for HbA1c, cLDL and blood pressure were more adherent to the respective drug classes, although there was not a statistically significant relationship between the control of those risk factors and therapeutic adherence.^19^ Ho et al.^15^ reported figures somewhat closer to ours, with 20%, 19% and 25% being nonadherent for OAHAs, antihypertensives and statins, respectively. However, the populations of these two latter studies greatly differ from ours. Lopez‐Simarro et al.^13^, ^19^ and Ho et al.^15^ included patients who were not necessarily exhibiting hypertension and dyslipidemia, and they could be receiving insulin. On the contrary, our population was much older (71 years) than that described by Lopez‐Simarro et al.^13^, ^19^ (68 years) and Ho et al.^15^ (66 and 62 years, for adherent and nonadherent patients, respectively). In another study conducted in the USA, which also included patients with diabetes, hypertension and dyslipidemia, nonadherence rates were similar to those reported by Lopez‐Simarro et al.^13^: 43% for metformin, 23% for ACE inhibitors and 36% for statins.^25^ Overall, we consider that it is likely that our study overestimated treatment adherence; the convenience sample as well as the requirement in our study that patients had to be receiving treatment for the three cardiometabolic risk factors for a minimum of 12 months could have contributed to these high rates of treatment adherence.

In their bivariate analysis, Ho et al.^15^ identified several factors associated with overall adherence, including male sex, older age and the presence of some comorbidities, such as hypertension, prior myocardial infarction, coronary artery disease and hypercholesterolemia. In our bivariate analysis, the presence of comorbidities as a whole was not associated with adherence, and according to the inclusion criteria, patients had to be receiving treatment for hypertension and dyslipidemia. However, the role of comorbidities in treatment adherence is unclear, and some authors have reported that in patients with diabetes, adherence to antihypertensives and lipid‐lowering agents is inversely associated with the number of prescriptions, and adherence to lipid‐lowering agents was also directly associated with the number of cardiometabolic conditions.^26^ In our study, there were no differences in age between adherent and nonadherent patients (71.4 vs. 71.9 years).

Despite good adherence as a whole and for the individual types of medication, only 31% of patients showed adequate control of the three cardiometabolic risk factors, increasing up to 36% when control of diabetes was defined based on individualized treatment targets. When evaluated individually, the proportion of patients with adequate control was much higher (73% for diabetes, 67% for hypertension and 64% for dyslipidemia). Consistent with what has been mentioned before for treatment adherence, in the study conducted by Lopez‐Simarro et al.^13^, ^19^ the corresponding figures for showing adequate control of the disease were 63% for diabetes, 41% for hypertension and 36% for dyslipidemia. Our results regarding overall disease control indicate that there is wide room for improvement in the management of patients with T2D who also exhibit hypertension and dyslipidemia. The control of these three risk factors is of paramount interest. The presence of comorbidities not only impairs quality of life and is associated with greater health care utilization^27^, ^28^ but, together with chronic kidney disease, comorbidities are also key contributors to mortality among patients with diabetes.^29^ It is also well known that targeting these multiple risk factors in patients with T2D is associated with a reduced risk of cardiovascular and microvascular events.^30^ In our multivariate analysis, global adherence was not associated with adequate control of the three cardiometabolic risk factors, and the only variable associated with adequate control was the presence of comorbidities, which was associated with a 57% reduction in the likelihood of those factors being adequately controlled. This finding is consistent with the previous results that found that some comorbidities are associated with poorer control of diabetes and other cardiometabolic risk factors.^31^, ^32^ Regarding the control of specific cardiometabolic risk factors, individual adherence to a specific type of medication was not associated with adequate control for the corresponding cardiometabolic risk factor. It is important to note that control of cardiometabolic factors is also multifactorial and not just dependent on adherence to medications. From this lack of association between adherence and control of risk factors arises the hypothesis that there is a third variable that could play a more important role than adherence in achieving good metabolic control, therapeutic inertia. The degree of control of cardiometabolic risk factors was consistently higher when it was based on physicians’ criteria than when it was based on the clinical practice guidelines, suggesting that physicians somewhat overestimate the effectiveness of their clinical and therapeutic recommendations, thus contributing to therapeutic inertia. In a study conducted by López‐Simarro et al.^19^ therapeutic inertia had a greater impact on BP and cLDL control than on the lack of adherence, whereas the control of glycaemia was influenced to a similar extent by the lack of adherence and therapeutic inertia. Nevertheless, it is important to acknowledge that therapeutic inertia could be clinically appropriate in a range of clinical situations such as the degree of patient frailty or limited life expectancy, as judged by the physician.^33^ Moreover, there are still many situations where, due to various circumstances such as competition from other demands, presenting borderline figures close to good control, lack of consultation time, diversity of recommendations between different clinical guidelines, pressures from health authorities to save costs, which mean that treatment is not intensified despite the health professionals recognize that this should be done.

In this study, we found that there was more therapeutic inertia and worse control for lipid‐lowering agents than for other therapeutic agents, although there are very efficacious drugs for the control of LDLc with very simple therapeutic regimens. Some factors that could have contributed to this result are the lack of consistency of some clinical practice guidelines and local institutions' recommendations regarding the treatment of dyslipidemia. Therefore, it is crucial that physicians be more aware of the importance of fighting therapeutic inertia and request periodic blood sample assays to review and reassess treatment for all their patients, even for those who are apparently well controlled.

Another interesting finding in this study is the importance of using individualized objectives for the control of HbA1c. This study suggests that when the HbA1c target is individualized according to patient characteristics, there is a higher proportion of patients with adequate control and much lower therapeutic inertia for the control of T2D.

Among the limitations of this study, we should mention its cross‐sectional design as well as the mean age of the patients (ie 71 years), since previous studies indicate that older patients with T2DM show better metabolic control than younger ones.^34^, ^35^ Despite we achieved the sample size estimated in the protocol for the assessment of our primary objective, our exploratory multivariate analysis regarding the adherence and other factors associated with the control of the cardiometabolic risk factors could be underpowered. Finally, it is also important to note that we did not record information on important socioeconomic factors (eg education and employment) that could have an impact on treatment adherence. In conclusion, despite relatively high adherence to all medications for treating T2D, hypertension and dyslipidemia, the control of cardiometabolic risk factors as a whole (namely T2D, hypertension and dyslipidemia) in the primary care setting in Spain is far from optimal. This could be related at least in part to the high comorbidity of these patients. Clinical practice guidelines for the management of diabetes should continue to stress the importance of targeting global metabolic control, which may be achieved by using individualized objectives for patients, with special attention to their comorbidities, and by addressing clinical inertia.

## CONFLICT OF INTEREST

DOB has received fees for lectures and participation in presentations from MSD, Sanofi, Novartis, NovoNordisk, AstraZeneca and Lilly and has served as a consultant to Sanofi. SCS received honoraria for lectures, participation in presentations and for the development of educational presentations from MSD, Boehringer‐Ingelheim, Almirall, Bristol Myers, Pfizer, Sanofi and AstraZeneca. JES has received consulting fees from MSD and Sanofi and for the development of educational presentations from MSD, Sanofi, Ferrer, Novartis, Lilly, Boehringer‐Ingelheim and Menarini. AGG, KFC, MCH and GF are full‐time employees at MSD Spain. FLS declares no conflict of interest in relation to this manuscript.

## AUTHOR CONTRIBUTIONS

**Domingo Orozco‐Beltrán:** Conceptualization (equal); Formal analysis (equal); Investigation (equal); Methodology (equal); Supervision (equal); Writing – original draft (equal). **Sergio Cinza Sanjurjo:** Investigation (equal); Supervision (equal); Writing – review & editing (equal). **José Escribano Serrano:** Investigation (equal); Supervision (equal); Writing – review & editing (equal). **Flora López Simarro:** Conceptualization (equal); Formal analysis (equal); Investigation (equal); Methodology (equal); Supervision (equal); Writing – review & editing (equal). **Gonzalo Fernández‐Zatarain:** Formal analysis (equal); Methodology (equal); Writing – review & editing (equal). **Antón Gómez‐García:** Formal analysis (equal); Methodology (equal); Project administration (equal); Writing – original draft (equal). **Karine Ferreira de Campos:** Formal analysis (equal); Methodology (equal); Supervision (equal); Writing – original draft (equal). **Marta Cedenilla Horcajuelo:** Conceptualization (equal); Formal analysis (equal); Methodology (equal); Project administration (equal); Writing – original draft (equal); Writing – review & editing (equal).

## CLINICAL TRIAL REGISTRATION

Not applicable.

## Data Availability

The data that support the findings of this study are available from the corresponding author upon reasonable request.
